# Comprehensive technology-assisted training and supervision program to enhance depression management in primary care in Santiago, Chile: study protocol for a cluster randomized controlled trial

**DOI:** 10.1186/s13063-015-0845-4

**Published:** 2015-07-24

**Authors:** Graciela Rojas, Pablo Martínez, Paul A. Vöhringer, Vania Martínez, Ariel Castro-Lara, Rosemarie Fritsch

**Affiliations:** Universidad de Chile, Departamento de Psiquiatría y Salud Mental, Clínica Psiquiátrica Universitaria, Av. La Paz 1003, Recoleta, Santiago, Chile; Núcleo Milenio de Intervención Psicológica y Cambio en Depresión, Av. Vicuña Mackenna 4860, Macul, Santiago, Chile; Universidad de Chile, Centro de Medicina Reproductiva y Desarrollo Integral del Adolescente, Facultad de Medicina, Universidad de Chile, Av. Profesor Zañartu 1030, Independencia, Santiago, Chile; Mood Disorders Program, Psychiatry Department, Tufts Medical Center Tufts University School of Medicine, Boston, Massachusetts USA

**Keywords:** Depression treatment, Primary care, Training and supervision program

## Abstract

**Background:**

Depression is a common and disabling condition. Since 2001, Chile has had a national program for depression in primary care and universal access to treatment for depressed people over the age of 15. There are National Guidelines to treat depression but no training program exists. The aim of the present study protocol is to measure the effectiveness of a comprehensive technology-assisted training and supervision program to enhance depression management in primary care.

**Methods and design:**

This is a two-arm, single-blind, cluster randomized controlled trial to compare the efficacy of the program versus usual care to treat depression in primary care clinics. In total, 434 depressed persons 18 to 65 years of age, recruited from four primary care clinics located in Santiago, will participate in the study.

**Discussion:**

In order to ensure the quality of interventions supported by the national program for depression in Chile, it is desirable to have training programs of proven effectiveness.

**Trial registration:**

NCT02232854, registered on 2 September 2014.

## Background

Worldwide, depression is one of the most common and disabling psychiatric disorders. By 2020, depression is projected to be the second highest cause of disability in both sexes. It is a public health problem as a result of its high prevalence, duration, and recurrence rates [1].

In Chile, prevalence studies carried out in the general population reported a point prevalence of 5.5 % for depressive episodes, according to the ICD-10 criteria [2], and a 6-month prevalence of 4.7 % for major depressive disorder [3]. Studies carried out in primary care reported prevalence rates of around 30 % [4, 5].

Depression can be treated effectively with low-cost antidepressants or psychological interventions. For people with persistent sub-threshold depressive symptoms or mild depression, low-intensity psychosocial interventions should be used, and for moderate or severe depression, a combination of antidepressant medication and a high-intensity psychological intervention are recommended. Stepped-care and collaborative models provide a framework for integration of drug and psychological treatments and help to improve rates of adherence to treatment [6, 7].

In Chile, a randomized controlled trial to treat depression demonstrated the effectiveness of a stepped care program in primary care clinics. The stepped care program included a structured psychoeducational group led by social workers and nurses, a systematic monitoring of clinical progress, and structured pharmacotherapy for patients with severe or persistent depression that was executed by trained general practitioners. The results showed a large and significant difference in favor of the stepped care program compared with the usual care across all assessed outcomes, and this difference remained stable over a period of 6 months. This program was more effective and only marginally more expensive than usual care for the treatment of depressed women in primary care. Women receiving the stepped-care program had an average of 50 additional depression-free days over 6 months relative to patients who received usual care [8, 9].

Since 2001, Chile has had a program for the detection, diagnosis, and treatment of depression in primary care clinics. It recommends algorithms for diagnostic evaluation, delivery of psychosocial interventions and medications, consultations with specialists, follow-up visits, and criteria for referral to secondary care. All persons with depression have to be treated in primary care unless they have psychosis, bipolar disorder, high suicidal risk, or a lack of response to two courses of treatment with two different antidepressants at therapeutic doses, plus psychosocial intervention [10].

In 2006, depression was incorporated into the national health reform, thus ensuring universal access to treatment for depressed individuals 15 and older [11], the majority of whom are treated by primary care teams [12]. Since 2009, the Ministry of Health has published two Clinical Guidelines for depression treatment, but no training program has yet been recommended [10, 13].

An evaluation of the depression program carried out at seven primary care clinics in two regions of Chile demonstrated that diagnoses of the severity of illnesses were inaccurate, that severity of depressive episodes was underestimated, that treatments were not indicated according to the severity of the illness or according to guidelines, that adherence to treatment was insufficient − higher for pharmacological treatment and lower for individual psychotherapy and group psychoeducation − and that patient dropout from the program was around 60 % [14, 15].

International evidence indicates that, despite the high prevalence of depression and availability of effective treatments, the diagnosis and treatment of depression by general practitioners do not follow clinical guidelines and that these guidelines do not always include training [16, 17].

Training can improve the detection, diagnosis, and treatment and could lead to better patient outcomes, although studies have shown that training alone does not result in improved outcomes [18, 19].

There is abundant literature on the effectiveness of collaborative care on depression [20]. Systematic literature reviews of strategies employed to improve mental health patient outcomes reported that the most effective interventions incorporate clinician education, nurse case management, a greater integration between primary and secondary care [21], and quality measures to improve results [22].

For these reasons, we believe that a comprehensive technology-assisted training and supervision program can improve depression management in primary care clinics in Santiago, Chile.

## Methods and design

This is a two-arm, single-blind (blinded only to outcome evaluator), cluster randomized controlled trial, which will compare the effectiveness of a comprehensive technology-assisted training and supervision program for depression management versus usual depression care in primary care clinics in Santiago, Chile.

### Aims and hypotheses

#### General aim

To carry out a cluster randomized controlled trial to compare the efficacy of a comprehensive technology-assisted training and supervision program versus usual care to treat depression in primary care clinics in Santiago, Chile.

### Specific aims

1. To compare the level of depressive symptoms of adults treated for depression through a comprehensive technology-assisted training and supervision program versus usual care in primary care clinics.
2. To compare patients’ adherence to a comprehensive technology-assisted training and supervision program versus usual care in primary care clinics.
3. To compare health-related quality of life of adults suffering depression treated in a comprehensive technology-assisted training and supervision program versus usual care in primary care clinics.
4. To compare clinical outcomes of patients treated in a comprehensive technology-assisted training and supervision program versus usual care in primary care clinics.
5. To compare use of health care services by patients treated in a comprehensive technology-assisted training and supervision program versus usual care in primary care clinics.
6. To compare the rate of treated depressed cases by teams participating in a comprehensive technology-assisted training and supervision program versus those following only the usual care approach in primary care clinics.

### Hypotheses

1. Adults receiving the intervention will reach clinical remission, given by a score of 10 or less in the PHQ-9, with a difference of 20 % when compared with the group receiving the usual care, 3 and 6 months after randomization.
2. Adults participating in the intervention program will have (significantly) better adherence to treatment than those receiving usual care, 3 and 6 months after randomization.
3. Adults participating in the intervention program will have significantly better quality of life than those receiving usual care, 3 and 6 months after randomization.
4. Adults participating in the intervention program will have significantly better clinical outcomes than those receiving usual care, 3 and 6 months after randomization.
5. Adults participating in the intervention program will have significantly greater service use than those receiving usual care, 3 and 6 months after randomization.
6. Primary care teams participating in the intervention program will treat more depressed cases than those that are only in the usual care program, during a 1-year timeframe.

### Setting and population

Adults between 18 and 65 years of age attending four primary care clinics located in two low-income municipalities of Santiago, Chile.

### Inclusion and exclusion criteria

Depressed adults aged between 18 and 65 years of age attending primary care clinics will be invited to participate. Informed consent will be obtained from each participant.

Depression will be assessed with the Patient Health Questionnaire-Nine items (PHQ-9) and the Mini-International Neuropsychiatric Interview (MINI). Individuals currently receiving treatment for depression and/or those without access to a telephone will be excluded.

### Intervention arm

The intervention arm includes a 16-h training program for primary care teams (physicians, psychologists, social workers, nurses, midwives, and occupational therapists) to ensure compliance with the Clinical Guidelines for the Treatment of Depression, issued by the Chilean Ministry of Health [10]. The workshop will include a seminar on the detection, diagnosis, and treatment of depression; discussion of clinical cases; and role-playing to enhance clinical skills and to apply problem-solving techniques. Finally, trained primary care teams will undergo an Objective Structured Clinical Examination (OSCE) for evaluation.

After the training, a focus group between primary health care teams and study researchers will be held in order to address barriers to the implementation of clinical guidelines. In order to develop a comprehensive supervision program, trained administrative staff will contact patients from a call center to support treatment adherence, and a psychiatrists will supervise the primary care team, using a web-based platform.

### Control arm

The control arm will receive treatment as usual − all the interventions that are guaranteed for persons with depression from the primary care clinics and if needed referral to the specialized psychiatric service, as indicated by the national depression program. Professionals will be told to follow the Clinical Guidelines for the Treatment of Depression [10].

### Outcome measures

#### Primary outcome measure

Depressive symptoms will be assessed as the primary outcome, using the Patient Health Questionnaire-9 item (PHQ-9) [23]. The PHQ-9 is a nine-item self-administered measure of depression, with documented reliability and validity in the sample population [24]. It is a dual-purpose instrument that, with the same nine items, can establish provisional depressive disorder diagnoses, as well as determine depressive symptom severity. Major depression is diagnosed if five or more of the nine depressive symptom criteria have been present at least “more than half the days” in the past 2 weeks, and one of the symptoms is depressed mood or anhedonia. As a severity measure, the PHQ-9 score ranges from 0 to 27 [23]. A score equal to 10 points or higher is indicative of major depressive syndrome. Severity can be categorized as follows: healthy (1–5), subclinical depressive symptoms (6–10), mild depression (11–15), moderate depression (16–20), and severe depression (21–27) [22]. In primary care studies, the questionnaire has a sensitivity for major depression of 88 %, specificity of 88 %, and positive likelihood ratio of 7.1 [23]. It was validated in a Chilean population [25].

### Secondary outcome measures

1. *Health-related quality of life* will be measured as a secondary outcome, using the Short Form-36 Health Survey (SF-36). It is a patient-reported and subjective measure of generic health-related quality of life [26, 27]. The SF-36 has eight subscales and two summary measures (physical and mental components), and each scale is transformed to a score from 0 (worse health status) to 100 (best health status). It has been validated [28] in Chile and has been widely used [29, 30].
2. *Clinical outcomes* will be measured using the Outcome Questionnaire (OQ 45.2). It is a self-administered, 45-item instrument that measures psychotherapy outcomes [31, 32], which has been validated in Chile and also widely used [33–35]. It considers three domains: symptoms and distress, interpersonal relationships, and social role.

### Other outcomes

Patients' adherence to treatment and use of health care services will be measured at 3 and 6 months after the baseline assessment. At the primary care team level, the rate of treated depressed cases will be measured 12 months before and after randomization.

### Sample size

Sample size calculation was done based on two studies previously carried out in Chile [36, 15]. A randomized clinical trial that compared pharmacotherapy monitored by telephone with usual care in primary care clinics in Chile found a recovery rate for depressive disorders of 62 % in the intervention group versus 38 % in the control group [36], having an effect size of 20 %. Since the current study protocol aims to test whether the intervention is better than usual care, a one-sided test is appropriate to detect an effect. Moreover, it is expected that the intervention will have a favorable effect ruling out the possibility of a statistically significant negative effect. To detect a difference of 20 %, in a one-sided model, with an alpha of 5 % and power of 80 %, 152 depressed persons, 76 in each group (intervention and control), will be required for the study. Using a database from another study comprising 201 persons treated for depression in seven primary care clinics [15], an intraclass correlation coefficient (ICC) of 0.03839 was estimated. Therefore, a design effect of 2.42 was determined, based on the previously calculated ICC and the use of four clusters (primary care clinics). After applying the design effect, the sample needed increased to 368 depressed persons. Considering a retention rate of approximately 85 %, 434 depressed cases will be required for the study, hence 109 cases in each of the four primary health care clinics.

Finally, a group of four primary health care clinics was chosen for two reasons: the usual flow of patients with depression presenting at each of the participating primary care clinics, which allows for a high number of potential participants per clinic, and financial constraints restricted the number of clusters, since having more primary care clinics would put in jeopardy the feasibility of the study.

### Recruitment

After being informed of the study by primary health care professionals, interested potential participants with possible cases of depression identified in routine clinical practice will be invited for an on-site or telephone evaluation by a study psychologist, who will obtain participants' informed consent, carry out a baseline evaluation and determine whether or not a potential participants fit study criteria.

### Group assignment

Clustered treatment assignment will be performed. First, two urban municipalities of the Metropolitan Region of Santiago, Chile, which collaborate with the Faculty of Medicine of Universidad de Chile and have at least two primary health care clinics, will be randomly selected through simple random sampling. Municipalities with a high Human Development Index, a high percentage of immigrants and older population, and psychiatry residents working in primary health care clinics will be excluded. In each selected municipality, the two primary health care clinics with the highest registered number of patients will be randomly assigned to the active or the control arm, through simple random sampling, thus selecting a total of four primary health care centers, one from the intervention arm and one from the control arm, in each selected municipality. This matching was done in order to ensure similarity between the patients’ socioeconomic levels and the administration and organization of the primary care centers, since all centers within a municipality are overseen by the same administration.

### Data collection

All participants will be assessed at baseline and at 3 and 6 months after recruitment. Independent interviewers blind to treatment assignment evaluated participants at 3 and 6 months after baseline assessment. The interviewers received an 8-h training to ensure standardization of data collection, which will be performed using digital PDF forms. Interviewers and patients will use computer tablets for data entry.

### Data management

Data collection is expected to be completed in late 2015. After each evaluation is complete, encrypted data entry forms will be sent by the interviewers via secure password-protected e-mail to the data management center. No copy of the data will remain on the computer tablets. Information will be compiled and exported to the CSV format for analysis in the SPSS software [37]. Only the principal investigator and co-investigator in charge of data management and analysis will have access to these data. Data will be encrypted and stored in a local hard drive. Subjects' identifying information will be eliminated from the database and preserved in a secure location. Data will only be identified by a unique ID code to ensure participants' anonymity.

### Data analysis

Data analysis will be performed using SPSS software [37], with which scores of the used scales will be calculated. Data and presentation of the results will be in accordance with CONSORT guideline for randomized clinical trials [38] as well as its extensions to cluster and non-pharmacological interventions, with the primary comparative analysis being conducted on an intention-to-treat basis. Initially, we will conduct descriptive analysis to assess the balance between the two groups. The primary analysis will employ multivariable linear regression to investigate differences in mean symptom scores (the primary outcome measure) between groups at 3 and 6 months after randomization, adjusting for baseline outcome variables if imbalances are identified. Sensitivity analysis based on different assumptions will be conducted to investigate the potential effects of missing data. Similar analysis will be carried out for secondary outcome measures.

### Monitoring

The primary care center staff will be told to report any type of adverse event (for example, hospitalization, pregnancy, suicide attempt) affecting the participants to the principal investigator, who will then take appropriate measures to ensure the safety and wellbeing of the participants.

### Research governance and ethics

#### Trial management

The study will comply with local research governance requirements.

### Ethics

Full ethical approval was obtained from the local Ethics Committee (Faculty of Medicine, Universidad de Chile, project no. 103–2012). Informed and written consent will be obtained from the participants.

## Discussion

To date, only a single study in Latin America has evaluated changes in the knowledge, attitudes, and practice among primary care physicians who participated in a depression training program, finding limited impact on daily practice a month after the training program, measured through rates of diagnosis, referrals, antidepressant prescription, and provision of supportive therapy [39].

In contrast to the study mentioned above, our research protocol aims to evaluate the effectiveness of a training program in improving patients' outcomes and focuses not only on training primary care physicians in managing depression, but also the primary health care team as a whole in order to develop a comprehensive approach to depression management, which is further reinforced by a technology-assisted supervision program.

The aforementioned makes our study the first of its kind in a Latin American country. We believe that the training and supervision program can contribute to the Chilean national depression program, which is implemented in all the primary care clinics of the country despite primary care teams not currently being trained to follow the Ministry of Health recommendations for the correct detection, diagnosis, and treatment of depression [10].

### Trial status

The study started recruiting patients in June 2014. Recruitment will end by July 2015.

## Abbreviations

ICD-10: 10th revision of the International Statistical Classification of Diseases
PHQ-9: Patient health questionnaire-9-item
MINI: MINI Mini-International neuropsychiatric interview
OSCE: Objective structured clinical examination
SF-36: Short form-36 health survey
OQ45.2: Outcome questionnaire 45.2
ICC: Intraclass correlation coefficient
PDF: Portable document format
CSV: Comma-separated values
SPSS: Statistical Package for the Social Sciences
